# Quantifying reciprocal relationships between poverty and health: combining a causal loop diagram with longitudinal structural equation modelling

**DOI:** 10.1186/s12939-024-02172-w

**Published:** 2024-05-01

**Authors:** Laurens Reumers, Niels Hameleers, Henk Hilderink, Marleen Bekker, Maria Jansen, Dirk Ruwaard

**Affiliations:** 1 Department of Health Services Research, Care and Public Health Research Institute (CAPHRI), Maastricht University, Maastricht, The Netherlands; 2 National Institute for Public Health and the Environment, Bilthoven, The Netherlands; 3 Chair Group Health and Society, Center for Space, Place and Society, Wageningen University and Research, Wageningen, The Netherlands; 4 Academic Collaborative Center for Public Health, Public Health Service South Limburg, Heerlen, The Netherlands

**Keywords:** Poverty, Income, Financial wealth, Social determinants of health, Quantification, Longitudinal, Structural equation modelling, Latent variables, Indirect effects, Causal loop diagram

## Abstract

**Background:**

This study takes on the challenge of quantifying a complex causal loop diagram describing how poverty and health affect each other, and does so using longitudinal data from The Netherlands. Furthermore, this paper elaborates on its methodological approach in order to facilitate replication and methodological advancement.

**Methods:**

After adapting a causal loop diagram that was built by stakeholders, a longitudinal structural equation modelling approach was used. A cross-lagged panel model with nine endogenous variables, of which two latent variables, and three time-invariant exogenous variables was constructed. With this model, directional effects are estimated in a Granger-causal manner, using data from 2015 to 2019. Both the direct effects (with a one-year lag) and total effects over multiple (up to eight) years were calculated. Five sensitivity analyses were conducted. Two of these focus on lower-income and lower-wealth individuals. The other three each added one exogenous variable: work status, level of education, and home ownership.

**Results:**

The effects of income and financial wealth on health are present, but are relatively weak for the overall population. Sensitivity analyses show that these effects are stronger for those with lower incomes or wealth. Physical capability does seem to have strong positive effects on both income and financial wealth. There are a number of other results as well, as the estimated models are extensive. Many of the estimated effects only become substantial after several years.

**Conclusions:**

Income and financial wealth appear to have limited effects on the health of the overall population of The Netherlands. However, there are indications that these effects may be stronger for individuals who are closer to the poverty threshold. Since the estimated effects of physical capability on income and financial wealth are more substantial, a broad recommendation would be that including physical capability in efforts that are aimed at improving income and financial wealth could be useful and effective. The methodological approach described in this paper could also be applied to other research settings or topics.

**Supplementary Information:**

The online version contains supplementary material available at 10.1186/s12939-024-02172-w.

## Background

### Introduction

The relationship between poverty and health has long been recognised. In 1924, a study on health differences between different groups of economic status states that “[t]hese data afford statistical evidence of what has in general been accepted, viz, that there is more sickness and a higher death rate among the poor than among the well-to-do” [1], p. 13. This indicates that as early as hundred years ago now, the existence of a relationship between poverty and health had been well-established.

In a broader context, poverty is regarded as one of the most prominent social determinants of health (SDoH) [2, 3]. SDoH are described by the World Health Organization as “the conditions in which people are born, grow, work, live, and age, and the wider set of forces and systems shaping the conditions of daily life” [4]. They are influential factors in shaping health outcomes [2, 5, 6], ubiquitous, and understanding them is therefore of great value for advancing public health. In order to study how SDoH affect health outcomes and how these outcomes may potentially be influenced, models containing causal mechanisms are warranted. Merely testing for correlations between a SDoH and health outcomes brings little understanding of what is actually happening and the correlations may largely be explained by confounders. Conversely, simply adding control variables to an analysis is likely to ‘close off’ (i.e. completely eliminate and disregard) mechanisms through which the SDoH affects health outcomes and therefore result in grossly underestimating effects [7]. Modelling the causal mechanisms of SDoH is difficult and subjected to two main challenges.

The first challenge is how to formulate a theoretical model that reflects expectations about real-world dynamics before moving towards quantification [8]. We addressed this first challenge in an earlier phase of this research project as we constructed a causal loop diagram (CLD) (an extensive, qualitative model of a system) using the participatory method of group model building with system dynamics [9]. The model from this study is focused on the relationships between poverty and health and provides the conceptual starting point for the present study.

The second challenge lies in quantitatively estimating the interrelationships between the variables from a CLD that depicts a complex system. In the present study, we wanted to know *how* SDoH can have an impact on health – and vice versa – through pathways involving multiple variables. As indicated, this was done using a CLD that was constructed in a previous study [9], but adaptations were needed for this model to be quantifiable. One difficulty in this is that the pathways between SDoH and health outcomes usually involve a number of interrelated variables and (sometimes ambiguous) relationships between those variables [10]. The complexity that results from having more than just a few (both independent and dependent) variables in a model – often with time lags [6] – makes quantifying them difficult. Such a complex set of interrelationships is one of the reasons why the ties between a SDoH and health outcomes can be considered to constitute a wicked problem [11]. The process of quantifying the interrelationships between poverty and health as modelled in the CLD in the previous study [9] is described in this paper.

The variables in the model will often not only affect other variables, but also indirectly influence themselves, which means that there is simultaneity. Simultaneity is understood as X influencing Y, while Y in return influences X [12], forming a feedback loop. Considering feedback loops is important for understanding how a system as a whole behaves over time and central to system dynamics [13]. For a qualitative conceptual model, this poses no fundamental methodological difficulties. However, for a quantitative model, such reciprocity violates the assumption of independence between the errors of the predictor and of the outcome variable [14] that statistical methods such as (multiple) regression depend on. Furthermore, all arrows in a CLD imply causal effects, so it is therefore crucial to use methods that can be used to make inferences about causal direction and not just show correlations.

The issues mentioned above can be addressed or circumvented using longitudinal structural equation modelling (SEM) [15]. SEM includes a range of methods suited for estimating complex model structures. While cross-sectional SEMs are, as noted by Uleman et al. [16], not suited to properly deal with feedback loops and with causal direction, longitudinal SEMs do incorporate temporal precedence and can circumvent those problems. This is done based on the logic that if B happens after A, it is not possible for B to cause A, which is further elaborated on in step 3 in the methods section of this paper.

The body of literature on the relationships between poverty and health is quite extensive.. The relationships between the financial side of socioeconomic position (SEP) and health have been the topic of a number of studies that use longitudinal SEM (e.g., [17–19]), and especially in the (health) economic literature there have been many studies estimating causal relationships between SEP and health using longitudinal designs (see e.g., [20] for an overview). Existing studies tend to focus on either one direction – the effect of SEP on health (behaviour) or vice versa (e.g., [21]), sometimes using instrumental variables (e.g., [22, 23]) – and usually focus on direct relationships between SEP and health without specifying mechanisms [24]. This is a suitable approach for studying to what extent the two affect each other. However, the goal of the present paper is also to elaborate on *how* poverty and health affect each other. In order to do this, mediating variables specifying mechanisms and a feedback loop structure can be used. This study demonstrates an approach that addresses both difficulties mentioned in this introduction section (obtaining a complex conceptual model and quantifying it) by combining system dynamics, longitudinal SEM, a number of variables in feedback loops, and mediation analysis.

#### Research question

The research question for this study is: *‘how and to what extent do poverty and health affect each other and are these findings in line with a CLD constructed by stakeholders?’* An additional aim of the study is providing an account on how this study’s methodological approach can be employed, which is intended as a starting point for others who might be interested in using it as well. While this study explicitly considers poverty and health, the approach itself is highly generally applicable and could be used for other SDoH as well and even outside of health-related research.

While many of these methodological elaborations are aimed at other researchers who are interested in quantification of SDoH, there are two other intended audiences for this paper. One audience consists of researchers who are not necessarily involved with quantitative research, but are interested in the topic of poverty and health, or SDoH in general. Most of this article will likely hold value for this group as well, though especially steps 3 and 4 of the Methods section may contain more information than is required. A final intended audience is that of policymakers. We think that especially Table 2 (including the extended one in Additional file 3) and the Discussion section contain insights that may be useful for this audience.

### Methods

In this section, we describe the basic steps that were taken and the challenges encountered during adapting and quantifying the CLD, and how various issues were resolved. Some of the more technical considerations that are part of the steps of this methodological approach can be found in Additional file 1.

#### Population and setting

**Fig. 1:**
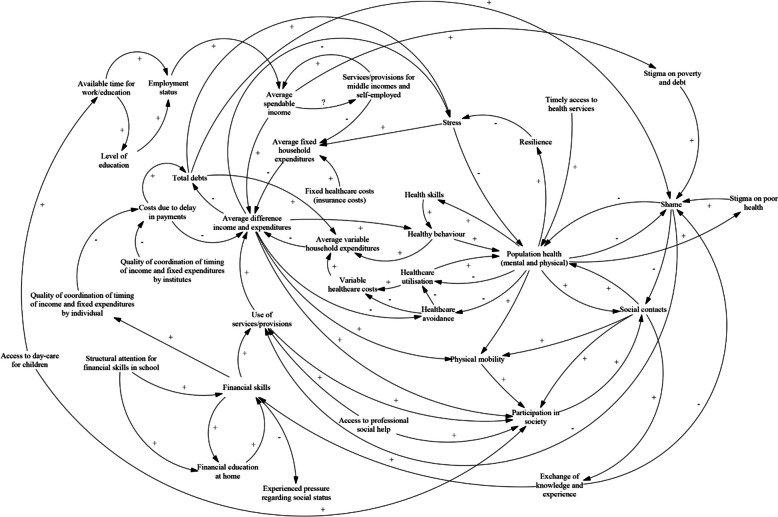
The causal loop diagram constructed using group model building in the previous study [9]

The setting in which this study is conducted is the country of The Netherlands and the population is defined as all inhabitants of The Netherlands. The CLD that is taken as conceptual model for quantification (Fig. 1) was built in the Dutch city of Utrecht, where there is particular regard for both poverty and health and involvement of stakeholders from societal practice in this process. The national level of analysis for quantification was chosen because of data availability and potential generalisability of results.

#### Data and sample

Individual-level data were used for quantification of the model, because the effects also occur on this level [25]. These data were derived from two datasets. The first of the two datasets is a large Dutch national survey, the Longitudinal Internet studies for the Social Sciences (LISS) panel, which contains data on thousands of individuals (exact sample size depends on the topics and wave selected – the health dataset from 2015 includes 6,009 respondents, for example) from all over The Netherlands [26]. A number of topics in this panel are relevant for this study, such as health, social integration and leisure, and other variables regarding personality and behaviour. Longitudinal datasets containing variables on all of these topics, which is essential for this study, are very scarce. For the original sample and first two replacement recruitments (2007–2012), random sampling was used. The third and fourth replacement recruitments (2013–2017) were drawn using stratified sampling, in an effort to maximise the sample’s representativeness. A comparison of population and sample characteristics is available [27]. The second dataset that is used is the official Dutch national registry data, managed by Statistics Netherlands (CBS) [28], which is used to complement the LISS survey data. These are data on all persons who officially live in the country, are longitudinal and cover the entire population with only little missing data. This data source was mainly used because it holds accurate and objective information on financial data, both on the individual and household levels.

The analyses in this study have been conducted using annual data from 2015 to 2019 from both datasets. For 2014 and several previous years, there were various crucial variables missing from the data, which is why 2015 was chosen as the first year for the analysis. The years 2020 and onwards were excluded from analysis because the outbreak of the coronavirus was assumed to influence the variables in the study in an idiosyncratic way that could not be controlled for. For an individual to be included in this study’s sample, just one of the questions on mental health or physical capability in any of the five included survey waves has to be answered *and* there has to be data on their financial position for at least one of those five years. No further exclusion criteria were applied. This resulted in a sample size of 6,581 individuals.

#### Steps to a quantified model

**Fig. 2:**
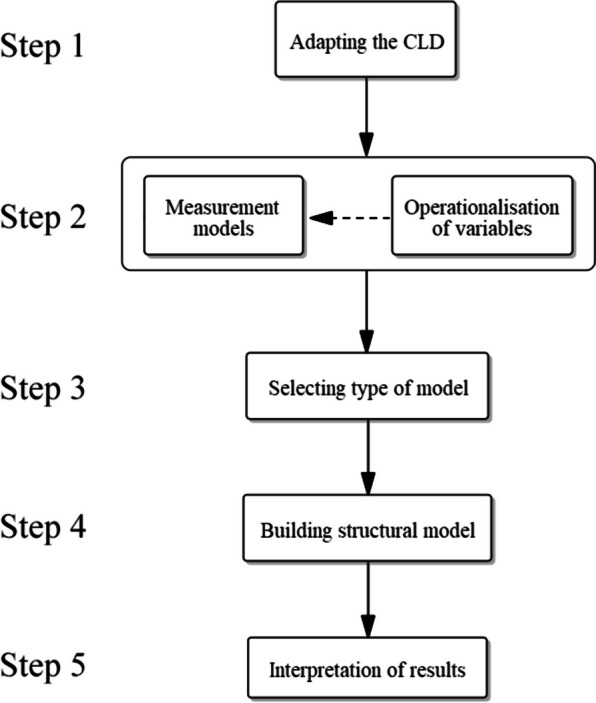
Flowchart showing the five steps of the quantification process

Quantification of the model can be roughly divided into five steps: adapting the CLD, measurement models and operationalisation, selecting the type of model, building the structural model, and interpretation of results. These steps were not as chronological and separated from each other as may seem from the descriptions below, but more of an iterative process with decisions made in later steps sometimes making it necessary to return to earlier ones to make adjustments. Constructing the measurement models and operationalising the variables were taken together as one step, as they are so interwoven that they were largely done at the same time. Figure 2 visualises the steps in a flowchart.

##### Adapting the CLD for quantification (step 1)

**Fig. 3:**
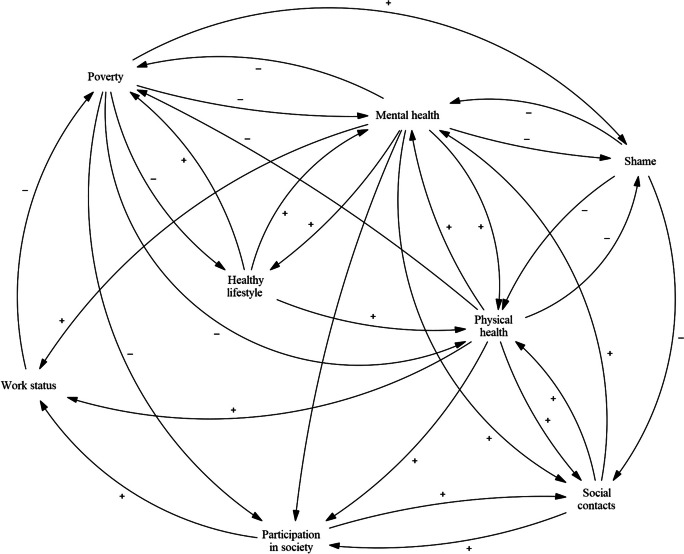
An adaptation of the causal loop diagram, based on comments from scientific experts and literature

The stakeholders’ CLD had to be simplified before quantification was possible. As there will be a limit to the number of variables that can be taken into account in such a quantified model, some decisions had to be made: which variables should be included and which are to be omitted, or merged, in favour of simplification? In the validation phase in the previous study, after the CLD was constructed by stakeholders who were heavily involved in practice and policy regarding poverty and health, a meeting with scientific experts was organised (see that study for more details [9]). In this meeting, experts were asked to highlight core elements of the model and to name any alterations they would expect to be necessary for the model to better reflect reality. Their input was helpful while adapting the model to make it quantifiable in the present study and led to making the distinction between physical and mental health. Furthermore, variables that were part of many and major feedback loops were given priority over variables that were not part of any or only ‘peripheral’ feedback loops. This led to a first version of a simplified CLD (Fig. 3). This version contains the variables of mental health, physical health, poverty, healthy behaviour, social contacts, participation in society, shame, and work status. The variable of stress was taken as being part of mental health – as stress is one of the items that will make up the latent variable of mental health, as will be further explained in the next two sections. The variables of home ownership and level of education were also mentioned by the experts as being potentially influential and are included in separate sensitivity analyses.

##### Measurement models and operationalisation of variables (step 2)

**Measurement models**

This study makes use of observed variables as well as latent constructs (in this paper, the terms latent variable, latent construct, and underlying construct are used interchangeably). Some variables are considered to be directly observable: the measurement is the actual value of the variable and not just an approximation of it. In this study, the variables age and sex could be regarded as being measured directly.

**Fig. 4:**
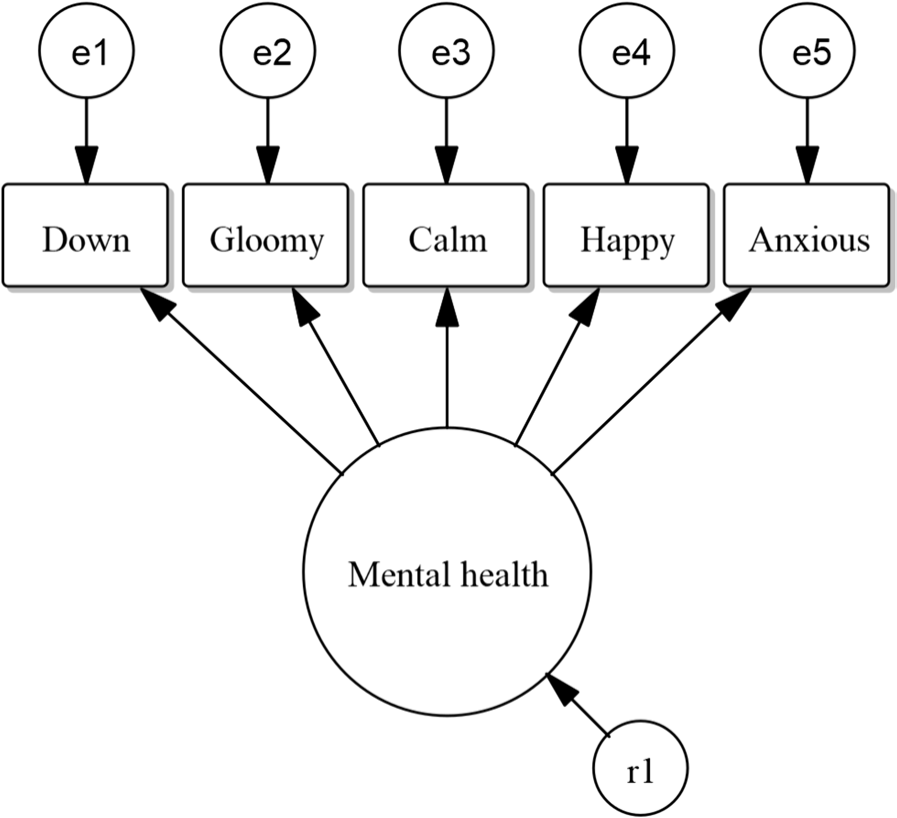
Measurement model (CFA) for latent variable mental health at one time point, using the five MHI-5 items [31]. The error terms of the indicators ‘e1’ to ‘e5’ are also shown, as well as the residual ‘r1’ for the latent variable

Latent constructs are variables that cannot be directly observed and measured, because they are comprised of more than is directly measurable. Mental health is an example of such a variable that cannot be captured in just one observed indicator; it contains multiple facets and is a complex construct. A one-item operationalisation might simply consist of a respondent’s self-rated mental health, but this is not a direct measurement of mental health itself. Neither are more specific survey items pertaining to the respondent’s mental health. Such operationalisations would be accompanied with fair amounts of measurement error [29]. This measurement error would attenuate the variable’s relationships in quantitative models, meaning that all estimates of effects tend to be biased – all effects would be weakened. By using multiple indicators together – indicators that are expected to be influenced by the latent variable – a more complete picture of the respondents overall mental health was given and measurement error removed. In this study, mental health and physical capability were included as latent variables, using confirmatory factor analysis (CFA) [30]. See Fig. 4 for a schematic depiction of the latent variable of mental health and Additional file 2 for a more detailed description of the operationalisation of these two latent variables.

In some other cases, more practical reasons led to the choice to not construct a latent variable for a particular variable. Acceptable multiple indicators measuring the variables of satisfaction with social contacts and satisfaction with leisure time were not found in the available data. Additional elaboration on what is required for multiple indicators to be deemed acceptable and reliable can be found in Additional file 1. For the construct of poverty, which theoretically and logically consists of multiple facets that do not indicate one common underlying construct, neither option (single item or latent variable) seemed acceptable. In response to this, both income and assets were included as single items measuring separate (observed) variables covering two different aspects of the construct. A person who has either a high income and few assets, or much financial wealth but little or no income, will normally not be seen as living in poverty. For this reason, the decision was made to include both variables in the model. Separating a construct into multiple single-item variables was also done for unhealthy behaviour. CFA showed that the available items were not suited for constructing one latent variable, and using a single observed variable would not be adequate either. Therefore, three separate observed variables were included as proxy variables for unhealthy behaviour: Body Mass Index (BMI, as a proxy for diet and physical activity), alcohol use, and smoking.

**Operationalisation of variables**

Below, brief descriptions of the items that were used for operationalising each variable are given. A more detailed overview can be found in Additional file 2. The items taken from LISS data are mental and physical health indicators, social contacts, health behaviour, and leisure time. The CBS data used were income, assets and debts, employment status, and migration status. The variables age and sex were operationalised using both LISS and CBS data.

Physical health was eventually included in the form of the more limited construct of physical capability (Additional file 1 details why this was done), meaning whether and how easily someone is capable of executing several specific physical tasks. For physical capability, four indicators relating to activities of daily living were used, with an ordinal scale from 0 (“not” [capable]) to 4 (“without any trouble”). These items measure the difficulty with which respondents can walk up a flight of stairs, wash or bathe themselves, walk 100 m, and carry a bag of 5 kg. Those four items were chosen because they are similar to items from the SF-36 questionnaire [32] and all have high factor loadings when put in a CFA, indicating that they measure the same underlying construct. The latent variable of mental health was measured with five observed variables, each with an ordinal scale from 0 to 5 (from “never” to “continuously", with the category reflecting the best mental health coded as 5). The five items that were used considered feeling down, gloominess, calmness, happiness, and anxiety – all five of the indicators used in the MHI-5 questionnaire [31]. As physical capability and mental health are both latent variables, they have no native scales. However, the observed indicators from which the latent variables are inferred, of course do have scales and the latent variables’ scales are derived from theirs [33] (see Additional file 1).

The variables of income and financial wealth are taken to be observable and measured using single indicators. For income, household income was adjusted for household size and composition – by using equivalence factors as provided by Statistics Netherlands [34] – and then expressed in percentages of the low-income threshold, which is corrected for inflation annually [35]. To illustrate, in 2015, the threshold was €1,030 for a one-person household and €1,930 for a household consisting of a couple with two children. Finally, these percentages were divided by 100 so that a score of 1 means an income of exactly one time the low-income threshold. Financial wealth was operationalised as total household assets minus household debts (*not* corrected for household size or composition) – excluding assets and debts relating to the respondent’s own home, as these are not directly spendable. It was then corrected for inflation, so that it is expressed in ‘2015 euros’. Then, it was divided by the median value for 2015, which is €21,414, so a value of 1 indicates one time the median household wealth of 2015. This was done to improve comparability and obtain similar standard error sizes across variables. Finally, in order to limit the impact of extreme outliers, cut-off points were placed at both plus and minus 50 times the median value, which limited the scores (and with that the impact) of the wealthiest individuals, fluctuating between 1.1 to 1.3 percent of the sample for each year.

For satisfaction with social contacts and satisfaction with leisure time, single-indicator measurements were used. Respondents were asked to indicate their satisfaction with both constructs on a scale of 0 (“not at all satisfied”) to 10 (“completely satisfied”). BMI was calculated using self-reported height and weight. Alcohol use was operationalised as the number of days per week a respondent drinks. Smoking was recoded into an ordinal variable with three categories: no smoking, smoking up to 5 units (cigarette, cigar, or pipe) per week, and smoking over 5 units per week.

All exogenous variables included in the models are time-invariant, which mean they are fixed and stay the same over the years. Operationalisations for these variables – age, sex, and migration background, and work status (also see point 4 of this section), level of education, and home ownership for the sensitivity analyses – can be found in Additional file 2.

**Fig. 5:**
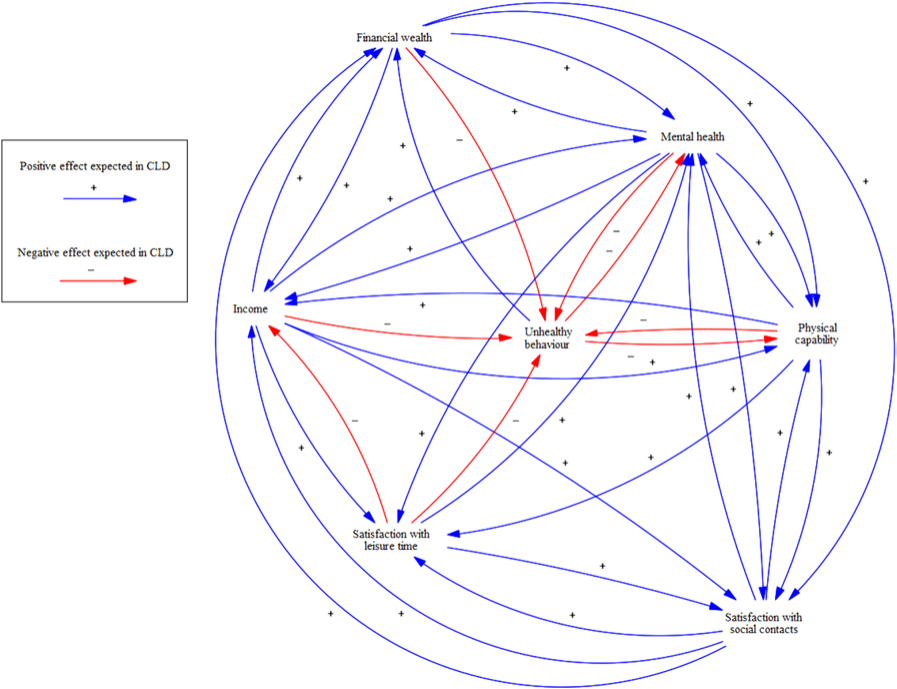
Final CLD structure with all variables and hypothesised effects that were eventually quantified. The three variables that are used to indicate unhealthy behaviour (BMI, alcohol use, and smoking) are not shown separately in this diagram for simplicity’s sake

At the end of the operationalisation phase, the model constructed in this study contains the variables mental health, physical capability, income, financial wealth, satisfaction with social contacts, satisfaction with leisure time, BMI, alcohol use, and smoking (see Fig. 5). The variables shame and participation in society were excluded, due to various issues. Participation in society has a conceptual overlap with the variables of satisfaction with social contacts and work status, which would have become problematic. Additionally, data availability was an issue for both shame and participation in society. Age, sex, and migration background were included as control variables.

##### Selecting the type of longitudinal SEM model and the estimator (step 3)

The type of SEM that was used in this study is a cross-lagged panel model (CLPM), following Orth et al.’s discussion on different types of longitudinal SEM and their different purposes and interpretations [36]. A CLPM considers both between-subject (i.e. between-person) and within-subject (i.e. within-person) variance together, which can be an advantage if one is interested in long-term effects. In addition, a CLPM can relatively easily capture the strength of each separate effect in a single coefficient, and can be used to cautiously model Granger-causal relationships. Granger-causality is based on the logic that because a variable logically cannot influence any variable’s past values, the directionality of effects can be ascertained, under the assumption that there are no omitted confounding variables [37]. Of course, there may still be omitted confounding variables, so an actual causal relationship between the variables cannot be ascertained. Lastly, the estimator selected for this study’s models is weighted least squares, which is suitable because of the presence of ordinal endogenous observed variables. More on choosing an estimators can be read in Additional file 1.

##### Building the structural model and invariance testing (step 4)

**Fig. 6:**
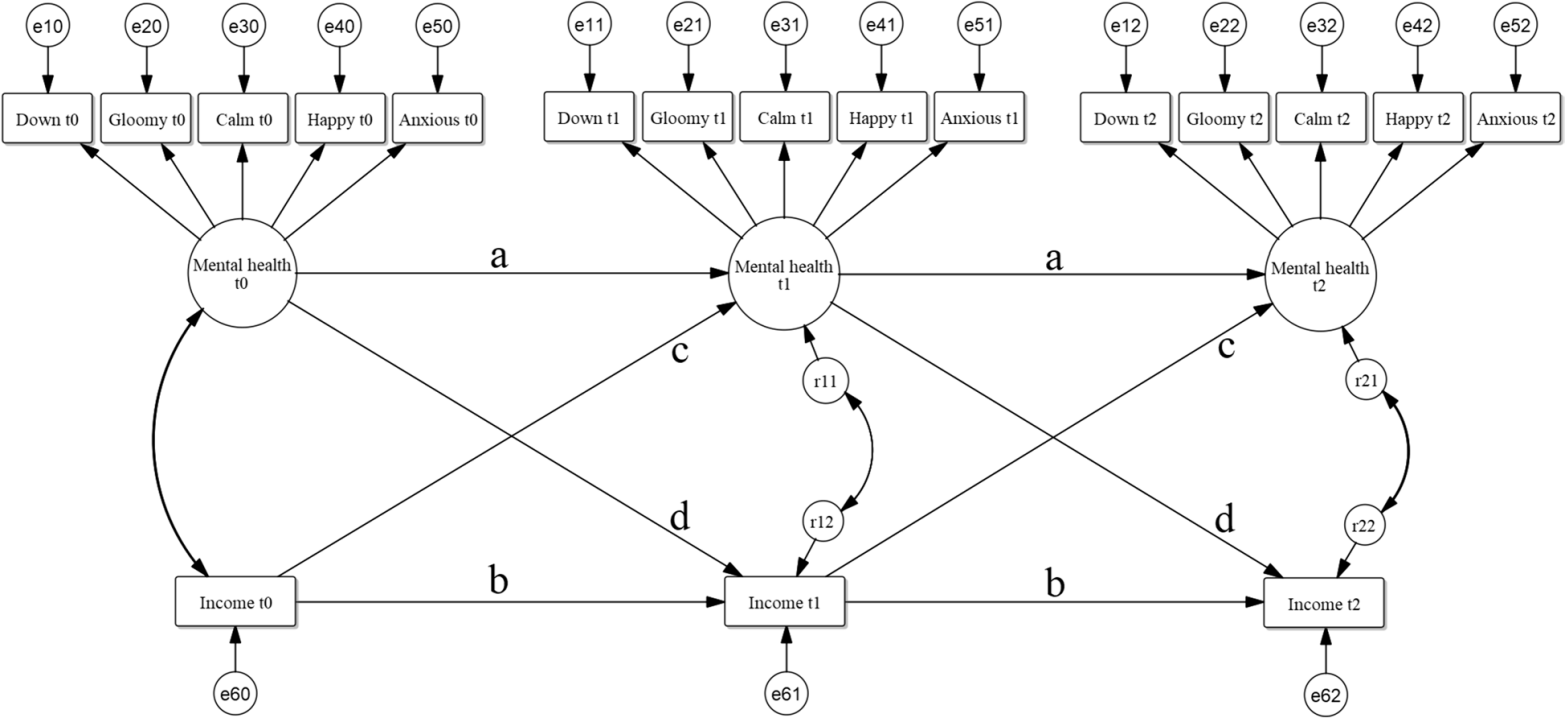
Two-variable CLPM structure with one latent variable and one observed continuous variable at three time points. The error terms (e) of the observed indicators are allowed to covary over time (not shown for simplicity’s sake). The residuals (r) are allowed to covary with the residuals of other variables at the same time point

A SEM can consist of two parts: a measurement model that constitutes any latent variables (and is optional) and a structural model that reflects how the variables in the model affect each other. The structural model in this study uses annual data, which means that every time interval has the length of one year. For instance, mental health in 2016 (t1) can be influenced by mental health in 2015 (t0) and income in 2015. Figure 6 shows a simplified diagram with just these two variables at three time points; the full model with all nine endogenous variables over five time points would be too large and cluttered to meaningfully depict.. The influence a variable has on its own subsequent value is the autoregression (*a* and *b* in Fig. 6); the influences it has on other variables are cross-lags (*c* and *d* in Fig. 6). All effects in the models in this study are entered as linear effects, in order to keep their complexity manageable, but it is possible to enter nonlinear effects into similar models. In Additional file 1, more elaboration on the CLPM structure and its components can be found. Lastly, there can still be confounders present in the model, which is the reason why the results can be interpreted as Granger-causal and not causal effects. If there are no confounders, it would be an actual causal effect, but this is not testable and probably not realistic in any real-world applications. As is the case with ordinary regression models, including variables in the model that are expected to influence a relationship of interest decreases the likelihood of important missing confounders. Since an aim of the stakeholders’ CLD was to attempt to identify and incorporate the most relevant variables on this topic, the SEM based on that CLD is likely to ‘control’ for at least some influential confounders.

Putting equality constraints over time on the regression coefficients (the relationships labelled with the same letter in Fig. 6.) did not decrease the fit of our model and were therefore retained. For the structural model, two further considerations are important: whether a variable is endogenous (dependent, or ‘Y’) or exogenous (independent, or ‘X’) and what the level of measurement of the variables is. All three of these topics are discussed in Additional file 1.

In addition, multiple sensitivity analyses were run. Their purpose is two-fold: they show whether effects are similar across models with different configurations of structural model or different subpopulations, and in doing so can also help shed some light on what may have caused results that were not the same as in the CLD. The primary model includes data on a sample drawn from the entire population of The Netherlands. It is however plausible that effects could be different for individuals in households with lower incomes or wealth. For this reason, separate analyses were run with two subsets of the sample. The first is the group whose household income is 2.5 times the low-income threshold (€30,900 for a one-person household in 2015) or less in at least two of the measurement years. The second group consists of those with a household financial net wealth (excluding worth of one’s home and mortgage) of two times the full sample’s median wealth (€41,828 in 2015) or less, in at least one measurement year. These thresholds are as low as they could be while retaining samples that are large enough to allow the model to be estimated.

In the CLD that was constructed by stakeholders, employment status was included as an exogenous variable (here meaning: not being part of the model’s feedback loops); whereas in the subsequent scientific expert meeting, the expectation was formulated that work status (having paid work) would have an important role in the system [9]. However, including it as an endogenous variable was problematic: due to its dichotomous nature, latent-response scales could not be linked over time. In order to do so, at least two thresholds would be needed [38], while a dichotomous variable has only one. Following the stakeholders’ CLD, a sensitivity analysis that includes work status as an exogenous and time-invariant variable was done. This was not done in the primary model, as the variable may well be influenced by the other variables in the model, as suggested in the scientific expert meeting [9]. If it is indeed part of any mediation mechanisms, it would not be truly exogenous, which could be problematic because it could largely ‘filter out’ effects where work status is a mediator [7]. However, the sensitivity analysis can be used to indicate possible confounding influences from work status. Two similar separate sensitivity analyses were then also conducted with the variables of level of education and home ownership added as exogenous variables, as a check to see how including them might affect the model. Information on the operationalisations of these variables can be found in Additional file 2. Finally, satisfaction with leisure time was included in the model partly because it was mentioned by the stakeholders building the CLD, but also because it may account for some of the (adverse) effects from work status.

##### Interpretation of the model (step 5)

After estimating the model, it is used for two purposes: interpreting the direct and indirect effects from one variable to other variables and testing the expected effects from the adapted CLD that was primarily based on expectations held by stakeholders (Fig. 5). Direct effects, the effects that were estimated in the SEM, exclusively consider a one-year time lag and no mediation via other variables in the model and therefore provide only a limited part of the overall picture. Including indirect effects enables the calculation of total effects (the sum of the direct effect and all indirect effects) from one variable on another, which are dependent on the length of the time frame that is selected. In this study, effects were calculated for until eight years after the model start point. Although it is possible to calculate effects beyond that, the assumption that extrapolation over time is indeed valid would get increasingly difficult to defend as the time frame gets longer. This is why the longest time frame used in this study is eight years, which is twice the interval between the first and last observed time point. Total effects are calculated for each permutation of variables. Additionally, this is done for the effect of a variable on itself at later time points. Effect sizes are shown using unstandardised and standardised coefficients.

Unstandardised (‘raw’) coefficients are expressed in their own units and are therefore not directly comparable to each other. These coefficients do, however, usually have real-world meaning: for instance, an increase of once the low-income threshold (€12,360 for a one-person household in 2015) could lead to an increase of β times the median wealth (€21,414 in 2015) in a subsequent year, where β is the unstandardised coefficient. In order to get a feeling for the magnitude of the effects, one has to keep in mind the units in which the variables are expressed. These can be found earlier in this methods section, under the sub-heading ‘Operationalisation of variables’, and in Additional file 2. Standardised coefficients are also reported, as is usual.

Using the results as a test of the a priori expected effects from the CLD is relatively more clear-cut than interpreting the coefficients by themselves, but still warrants attention. A CLD is constructed having direct effects in mind, so a comparison between the CLD and direct effects is in order. However, as described earlier, total effects show more of the full picture than direct effects do, so it seems useful to also involve these in the interpretation. Additionally, there are unstandardised and standardised coefficients that do not necessarily produce the same qualitative conclusions, and five sensitivity analyses were also run in this study. We opted to compare the CLD with expected relationships (Fig. 5) to unstandardised and standardised total effects from all six models and to the standardised direct effects from the primary model. This is done in the next section.

### Results

#### Total effects between the main variables of interest

The primary model and all sensitivity models were estimated for a one-year lag (the direct effects) up to an eight-year lag. The primary model indicates good fit (robust model fit indices: CFI 0.966, TLI 0.966, RMSEA 0.037, SRMR 0.046), as do the sensitivity analyses (fit indices available in Additional file 3). In the text below, unstandardised and standardised regression coefficients from this model are presented. For brevity’s sake, this is done only for the lag that shows the strongest effect, but for the main variables of interest, the full results from the primary model with comparison to expectations are shown in Table 1. A summary of effects between these variables from the primary model and the five sensitivity analyses can be found in Table 2. The results from all models for all estimated effects and model fit indices are provided in Additional file 3. The raw *lavaan* (the modelling software package used in this study [39, 40]) output for the primary model can be found in Additional file 4, and the polychoric correlation matrix for all observed variables in this model in Additional file 5.

**Table 1:** Unstandardised and standardised total effect coefficients from the primary model calculated for eight years, with comparison to expectations from the CLD (*N* = 6,581; df = 3,154; CFI = .991; TLI = .991; RMSEA = .020; SRMR = .046)

		Unstandardised effect coefficients				Standardised effect coefficients											
**X (predictor)**	**Y (outcome)**	**1**	**2**	**3**	**4**	**5**	**6**	**7**	**8**	**1**	**2**	**3**	**4**	**5**	**6**	**7**	**8**
*Mental health*	*Mental health*	*.833*	*.699*	*.591*	*.502*	*.428*	*.367*	*.316*	*.273*	*.833*	*.699*	*.591*	*.502*	*.428*	*.367*	*.316*	*.273*
Physical capability	Mental health	.047	.077	.097	.110	.119	.125	.129	.131	.052^a^	.086^a^	.107^b^	.122^b^	.132^b^	.138^b^	.142^b^	.145^b^
Income	Mental health	.004	.012	.019	.026	.031	.035	.037	.039	.005	.013	.022	.030	.036	.040	.043	.045
Financial wealth	Mental health	.001	.002	.001	.001	.001	.000	.000	.000	.012	.013	.011	.008	.006	.004	.003	.003
*Physical capability*	*Physical capability*	*.919*	*.860*	*.814*	*.776*	*.744*	*.715*	*.690*	*.667*	*.919*	*.860*	*.814*	*.776*	*.744*	*.715*	*.690*	*.667*
Mental health	Physical capability	.038	.061	.075	.083	.087	.089	.089	.088	.035	.055^a^	.068^a^	.075^a^	.079^a^	.081^a^	.081^a^	.080^a^
Income	Physical capability	.015	.019	.020	.018	.015	.012	.009	.007	.015	.020	.020	.018	.016	.013	.010	.007
Financial wealth	Physical capability	-.001	.000	.002	.004	.007	.010	.012	.015	-.009	-.001	.015	.034	.054^a^	.074^a^	.094^a^	.112^b^
*Income*	*Income*	*.868*	*.755*	*.659*	*.575*	*.502*	*.438*	*.383*	*.334*	*.868*	*.755*	*.659*	*.575*	*.502*	*.438*	*.383*	*.334*
Mental health	Income	.000	.003	.006	.009	.011	.014	.015	.016	.000	.002	.005	.008	.010	.012	.013	.014
Physical capability	Income	.046	.082	.111	.135	.155	.172	.187	.199	.044	.078^a^	.106^b^	.129^b^	.149^b^	.165^b^	.179^b^	.191^b^
Financial wealth	Income	.004	.006	.009	.011	.012	.014	.015	.017	.027	.047	.064^a^	.078^a^	.091^a^	.102^b^	.112^b^	.122^b^
*Financial wealth*	*Financial wealth*	*.916*	*.866*	*.835*	*.814*	*.800*	*.789*	*.781*	*.774*	*.916*	*.866*	*.835*	*.814*	*.800*	*.789*	*.781*	*.774*
Mental health	Financial wealth	-.020	-.112	-.222	-.326	-.416	-.488	-.545	-.587	-.002	-.013	-.026	-.038	-.049	-.057^d^	-.064^d^	-.069^d^
Physical capability	Financial wealth	.000	.188	.456	.754	1.054	1.345	1.624	1.888	.000	.024	.059^a^	.098^a^	.137^b^	.175^b^	.212^c^	.246^c^
Income	Financial wealth	.183	.239	.233	.197	.149	.096	.043	-.009	.025	.032	.032	.027	.020	.013	.006	-.001

Legend for standardised effect coefficients:

^a^Small effect: .050 to .100, sign expected

^b^Modest effect: .100 to .200, sign expected

^c^Decent effect: .200 to .300, sign expected

^d^Small effect: -.100 to -.050, sign unexpected

*Autoregression, *sign self-evident

**Table 2:** Summary of standardised total effects from all models at their peaks within eight years, with comparison to expectations from the CLD

X (predictor variable)	Y (outcome variable)	Primary model	Sensitivity analyses				
			**Low income**	**Low wealth**	**Work status**	**Education**	**Home ownership**
Physical capability	Mental health	** + + **	** + + **	** + + **	** + + **	** + + **	** + + **
Income	Mental health		** + **				
Financial wealth	Mental health						
Mental health	Physical capability	** + **	** + + **	** + + **	** + **	** + **	** + + **
Income	Physical capability			** + **	** + **		** + **
Financial wealth	Physical capability	** + + **	** + **	** + **			
Mental health	Income						
Physical capability	Income	** + + **	** + + **	** + + **	** + + **	** + + **	** + + **
Financial wealth	Income	** + + **	** + + **	-*	** + + **	** + + **	** + + **
Mental health	Financial wealth	-*	-*	- -*			
Physical capability	Financial wealth	** + + **	** + + **	** + **	** + **	** + + **	** + + **
Income	Financial wealth			** + **			

**+ + **Decidedly positive: .100 or higher, sign of effect was expected

**+ **Positive: .050 to .100, sign of effect was expected

**- - **Decidedly negative: -.100 or lower, sign of effect was expected

**- **Negative: -.100 to -.050, sign of effect was expected

- -* Decidedly negative: -.100 or lower, sign of effect was unexpected

-* Negative: -.100 to -.050, sign of effect was unexpected

Mental health in this model is clearly affected by physical capability. The (unstandardised and standardised) effect of physical capability on mental health increases steadily over time and is strongest after eight years (.131 and .145). This indicates that a one-unit increase in physical capability leads to an increase of .131 points on the latent mental health variable. Income has a smaller effect on mental health, with .039 and .045 after eight years. The unstandardised effect is over twice as strong for the lower-income group (.082), but the standardised effect is similar (0.054) to that in the primary model. The effect of financial wealth on mental health is very small (.002 and .013 after two years).

Physical capability in turn is influenced by mental health, which has a modest but clear effect on it, with .089 and .081 after seven years. The effect of income on physical capability is small in the primary model (.020 and .020 after three years). It is much stronger in three sensitivity analyses, especially the analysis with only the lower financial wealth group (.091 and .079 after eight years). The unstandardised effect of financial wealth on physical capability is small, but seems much larger when standardised (.015 and .112 after eight years). The latter is likely a result of the much larger standard deviation of financial wealth, which illustrates why it is generally advisable to not only interpret standardised effects.

Income is most strongly affected by physical capability, with an effect of .199 and .191 after eight years. Mental health displays a small effect on income (.016 and .014 after eight years). In the analysis with only the lower financial wealth group, this effect is a little over twice as strong (.038 and .040 after eight years). For financial wealth on income, the unstandardised coefficient again is small, but the standardised coefficient more substantial (.122) – once more a coefficient that is likely inflated by the larger standard deviation of financial wealth.

Financial wealth displays a prominent influence from physical capability, even though the direct effect was not modelled, with coefficients of 1.888 and .246 after eight years. This effect is decidedly less prominent in some of the sensitivity analyses, but is still strong in all of them. Mental health has a negative effect on financial wealth of -.587 and -.069 after eight years. This is the only one of the effects between the four main variables that shows the opposite of what was expected beforehand. The sensitivity analyses all show a moderately to almost entirely diminished effect (though it does remain negative in all of them), which suggests that this unexpected effect may largely be explained by the elements added in these analyses, such as work or education. Lastly, for the effect of income on financial wealth, the coefficients are .239 and .032 after two years, after which it shows a clear decrease to almost zero after eight years. This suggests short-term returns which are cancelled out in the long run. This decrease is much less strong in nearly all of the sensitivity analyses.

#### Notable total effects of and on other variables

Some of the effects involving other variables in the model also warrant being mentioned, because they are either strongly in line or in contradiction with the formulated expectations. In the primary model, effects that are clearly in line with the expectations are those of physical capability on smoking (negative), smoking on physical capability and on financial wealth (both negative). The results from the primary model seem to contradict expectations in the effects of income and mental health on smoking (both positive) and of BMI on smoking and alcohol use (both negative). Not all of these effects are the same in all of the sensitivity analyses. More detailed model results can be found in Additional file 3.

#### Comparing standardised direct effects to expectations

**Fig. 7:**
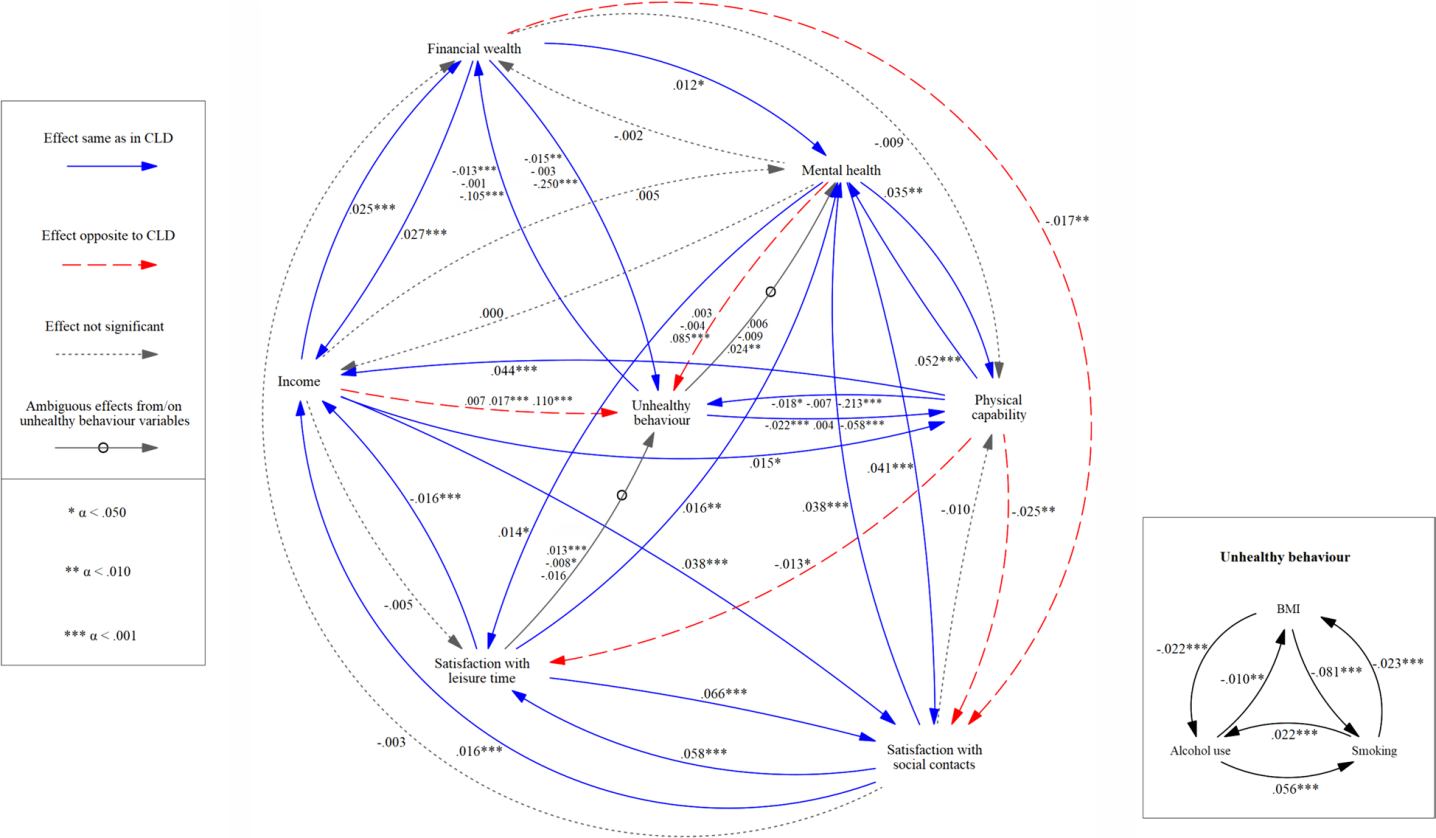
Model with standardised direct effects from the primary model. The three coefficients accompanying every arrow from and to unhealthy behaviour indicate individual relationships with BMI, alcohol use, and smoking, in that order. There were no predictions in the CLD regarding the relationships between these variables making up unhealthy behaviour (the box in the bottom right corner), so these were not compared to any prior expectations

In the adapted CLD, 50 direct effects were predicted (treating BMI, alcohol use, and smoking as separate variables). The standardised estimates for these effects are shown in Fig. 7, along with the effects between the variables making up ‘unhealthy behaviour’. For clarity’s sake, only the endogenous variables are shown in this figure. Of the 50 predicted effects, 27 effects are significant and in line with prior expectations, 17 are non-significant, and six are significant and opposite to expectations.

Four variables and their effects on each other were found to be crucial in the interrelationships between poverty and health: mental health, physical capability, income, and financial wealth. Of the 11 expected effects between these variables, seven are significant and in line with expectations and four are non-significant. Between mental health, physical capability, income, and financial wealth, only five of a possible 11 direct effects are significant (and all in line with expectations). These are the effects of mental health on physical capability and vice versa, physical capability on income and vice versa, and financial wealth on mental health. A possible direct effect of physical capability on financial wealth was not included in the original CLD and therefore not estimated.

### Discussion

#### Summary of results

Regarding the research question – *‘how and to what extent do poverty and health affect each other and are these findings in line with a CLD constructed by stakeholders?’* – there are two clear insights that can be highlighted in summary. First, improving physical capability seems to be very beneficial to both financial wealth and income, as well as to mental health and healthy behaviour. Second, income and especially financial wealth seem to have limited effects on the health of the *overall* population. However, there are indications that income and financial wealth are more important for the health of people in lower-income and lower-wealth groups and their effects may be even stronger for people living in poverty.

#### Interpretation of model results

In the primary model, most effects between mental health, physical capability, income, and financial wealth are modest to small. The clearest exceptions to this are the effects from physical capability on the other three variables, though one should keep in mind that a change of 1 unit on the scale of physical capability is a relatively large change due to its small standard deviation. Nevertheless, a one-unit change of physical capability leads to a predicted .12 unit increase in mental health, a .20 unit increase in income (approximately €2,500 annually for a one-person household) and a 1.9 unit increase in financial wealth after eight years (approximately €43,000 for a household), which seems substantial. All of these effects remain substantial in all sensitivity analyses. This suggests that improving physical capability may be an effective way to improve both mental health as well as income and financial wealth. The effects of income on financial wealth and that of mental health on physical capability are also relatively substantial and stable, and in line with expectations.

Conversely, among the main variables of interest, there are two results from the primary model that were unexpected based on the CLD. First, the estimated effects of income and financial wealth on the health variables in the population as a whole are smaller than one might expect. The effect of income on mental health is much more strongly positive in the lower-income group; the effect of income on physical capability is also much stronger in the model with only the lower-income group but especially in that with only the lower-wealth group. These findings indicate that income may not be too consequential in determining the mental health and physical capability of the well-to-do part of the population, but is important for those who have a lower income or less financial wealth. Money may simply have more value for health per euro for these groups. Unlike the effects of income, the effect of financial wealth on mental health and physical capability is small in all models, and only the effect on physical capability in the standardised model seems substantial. It could be that this is different for even more specific groups, for example those with an income below the low-income threshold, but this was not tested in this study. Second, the negative effect of mental health on financial wealth is the opposite of what was expected based on the CLD. However, this effect has mostly dissipated in the sensitivity analysis that includes work status as exogenous variable, suggesting that factors having to do with having work or experiencing work pressure may play some confounding role here.

#### Strengths and limitations

This study addresses the question of how poverty and health are interrelated. In doing so, it introduces a methodological approach that combines four elements that are not usually used in conjunction: a CLD built by stakeholders, a longitudinal SEM design, a large number of endogenous variables with reciprocity, and mediation analysis. The approach entails a chain of decisions addressing methodological challenges in data and analysis. Estimating a complex system of endogenous variables has two main benefits: it generates more detailed insights about relationships that include mediation mechanisms and it removes confounding effects of a number of variables without eliminating mechanisms in which these variables are mediators. Doing so does require a conceptual model of relevant variables and how these are connected with each other. Using a causal loop diagram as qualitative input for the structure of the model and a set of expectations provides a non-arbitrary starting point from which to test these expectations [9]. Additionally, the longitudinal SEM design has multiple advantages. SEM allows the use of latent variables and for endogenous variables to also serve as mediators. A *longitudinal* SEM makes it possible to cautiously infer directionality, in a Granger-causal [37] way. It permits one to draw conclusions such as that, assuming no unmeasured confounders, the effect of physical capability on financial wealth is clear and strong, while the effect of financial wealth on physical capability is quite small. Moreover, the models resulting from this study can be used to make multi-year projections – with and without external interventions – thereby informing decision-making processes. Such projections can be tailored to fit specific (sub)populations simply by using aggregated-level population characteristics, where available.

There are of course also multiple considerations, assumptions, and limitations present in the study and the chosen design. Many choices are necessarily made during the entire modelling process, from the start of the construction of the CLD to the final quantification steps. A first limitation relates to the construction of the CLD and is addressed at length in the article that describes that previous part of the process [9]. In short, it entails that the CLD was built by a limited group of participating stakeholders (although with experience in practice and policy) within a specific setting (the city of Utrecht). While this is a non-arbitrary way to obtain a conceptual model, different participants or a different setting could yield a different model. Rather than introducing any model as *the* model of the issue, it should be presented as being *a* model. An important advantage of using SEM is that the conceptual model as a whole can be tested empirically, using model fit indices. However, this does not mean that it can be tested whether a model is ‘true’ or not – only how well the model structure fits the real-world data [41].

The second limitation concerns issues related to the operationalisations of physical capability and of poverty. After consideration, physical capability was included as a variable that represents a part of physical health. It has a more limited scope than physical health in general and for this reason may produce different results than broader operationalisations would. The concept of poverty was captured by considering both income and financial wealth and does not include household expenditures. The latter are more difficult to operationalise. Additionally, higher-income households might have higher expenditures as well. A household with much financial wealth and/or a very high income that chooses to spend more than their income can hardly be said to be living in poverty. Having higher expenditures or even a negative income-expenditures balance would therefore not necessarily be a good indicator for poverty, although these factors can contribute to perceived income inadequacy, which is reported to have health effects as well [28]. To retain as much information as possible, income and financial wealth were included as continuous variables.

That brings us to a third limitation: besides effects having been constrained in several other ways, they are also assumed to be linear. This means that the effect of X on Y is the same for each value of X, so a euro of income is modelled to matter as much to someone who earns €20,000 euros a year as it does to someone who earns €200,000. This assumption is necessary to avoid unmanageable model complexity, but it is not always equally plausible. By also running sensitivity analyses for only lower-income and lower-wealth groups, the moderating effect of having a low income (as a dichotomous property) on the effects can be shown. This provides insight into how effects are different for these groups, such as that income seems to have effects on both mental health and physical capability that are approximately twice as strong for the lower-income group. Such sensitivity analyses however do not show the direct effects of the dichotomous low-income variable on the other variables. Alternatively, differences between these two sensitivity models and the primary model can be interpreted as showing nonlinearity in the effects of income and wealth on other variables. Something that should also be noted is that in order to make the selection for the lower-income and lower-wealth groups for the sensitivity analyses in this study, cut-off points were set at 2.5 times the low-income threshold and two times the sample’s median wealth, respectively. These cut-off points could not be set lower in order to maintain a sufficient sample to estimate the model, but a model in which it is possible to narrow down the groups further might potentially produce stronger effects still. Finally, it seems likely that at a high enough income level, any further increases in income no longer lead to significant improvements in health. The results from this study reflect a diminishing incremental effect of income on mental health, but do not show how the effect’s gradient is shaped. It could be that the effect gradually diminishes, but it is similarly possible that there are threshold effects present. This applies to any of the relationships in the model, as the model was built with the assumption of linear effects. Studying incrementally diminishing effects in depth was not a main focus of this study, but could be done by constructing models that use an extensive multiple group or nonlinear design.

A fourth limitation is the possibility of unmeasured confounding effects. To counter these, this study includes a good number of relevant endogenous variables. By including them as endogenous, they are not merely ‘controlled for’ but can actively contribute in mediation mechanisms. The variable of work status was a particularly challenging variable. The stakeholders’ CLD and the participants from the scientific expert meeting included the variable as exogenous and endogenous, respectively. The dichotomous nature of the variable made it impossible to test the assumption of threshold invariance over time [38] and to include it in the model as varying over time (as would be necessary for a endogenous variable). At the same time, including it as exogenous in the primary model would be problematic if it was in reality endogenous. Therefore, it was included as a time-invariant exogenous variable in a sensitivity analysis, in order to examine possible effects that this variable has on the rest of the model, as was also done for level of education and home ownership. This is a limitation to the results of the study, however, and some mediation effects may be ‘filtered out’ of the results*.* Furthermore, the primary model and the sensitivity analyses show multiple alternatives with good and similar model fit (see Additional file 3) and this may create uncertainty regarding which one is the ‘best one’. The short answer is that this depends on the intended purpose. The primary model is most suited for application to the population as a whole, while the models focused on the lower-income and lower-wealth groups are intended to provide information on a more specific target group. It should be noted that the results from the model with only the lower-wealth group strikingly deviate from all the other models. This could be due to actual and clear differences between the groups, but it may be wise to approach this model more cautiously until there is more evidence to support its claims. Lastly, the models that control for work status, level of education, and home ownership remove these variables as possible unmeasured confounders. Conversely, relationships in which those variables are part of the mechanism itself (as mediator) are ‘closed off’, so are likely to be underestimated. This should be kept in mind when using one of these models.

A fifth and final limitation is that the specific model design may influence findings, in that a CLPM requires the specification of the length of the time lag to be estimated. In this study, this time lag had the length of one year. However, it is possible for this lag to be longer or shorter in reality. Any relationships that have an actual time lag that is in reality (much) longer than this, may have been underestimated. Conversely, if the actual lag is shorter, data points closer to each other would be needed to detect effects. Effects with a shorter-term lag than is modelled are also potentially underestimated, which would make the results presented in this study more likely to be conservative estimates than inflated ones.

### Conclusions

In short, several key findings can be summarised from this study. There is an indication that physical capability (but not mental health) may have a substantial influence on income and financial wealth, and also on mental health. Financial wealth has some effect on physical capability in the models, but not on mental health. Income does not show a clear effect on either mental health or physical capability for the general population, but there are indications that income and financial wealth are more important for the health of lower-income and lower-wealth groups and their effects may be even stronger for people living in poverty. A broad recommendation from this study would be that physical capability is also considered in activities that are intended to improve income, financial wealth, or mental health.

Regarding the methodological approach, there are lessons to be drawn from this study as well. A positive one is that the quantification of a CLD can be done using longitudinal SEM, even while preserving a large part of the complexity and the feedback loops. Naturally, there are a number of assumptions and limitations inherent to achieving this. In the end, a model is always a simplified approximation of reality and is therefore ‘wrong’ by default [42]. However, the models from this study have been able to show quantitative estimations and generate insights and evidence that would otherwise not have been found, providing an indication of how mechanisms between poverty and health may operate.

### Supplementary Information

**Supplementary Material 1. **

**Supplementary Material 2. **

**Supplementary Material 3. **

**Supplementary Material 4. **

**Supplementary Material 5. **

## Data Availability

Any requests for additional information regarding this study can be addressed to the corresponding author. LISS data is free and can be accessed for non-commercial research. The CBS data that was used is non-public microdata. Under certain conditions, these microdata are accessible for statistical and scientific research. For further information: microdata@cbs.nl. The software used for the estimation of structural equation models in this paper was the R package ‘lavaan’ (version 0.6–5; R version 4.2.3). Data transformations were done using SPSS version 25. The figures shown in this paper (Figs. 1, 2, 3, 4, 5, 6 and 7) were made in Vensim PLE version 9.3.5 (developed by Ventana Systems), which is available for Windows and Macintosh OS (X) from https://vensim.com. This version of Vensim is free for academic use, but requires a paid license for non-academic use.

## Abbreviations

SDoH: Social determinant(s) of health
CLD: Causal loop diagram
SEM: Structural equation model(ling)
LISS: Longitudinal Internet studies for the Social Sciences
CBS: *Central Bureau voor de Statistiek* (Statistics Netherlands)
CFA: Confirmatory factor analysis
MHI-5: Mental Health Inventory 5
BMI: Body Mass Index
CLPM: Cross-lagged panel model
CFI: Comparative fit index
TLI: Tucker-Lewis index
RMSEA: Root mean square error of approximation
SRMR: Standardised root mean square residual
df: Degrees of freedom
