# Supplementary material for: Quantifying reciprocal relationships between poverty and health: combining a causal loop diagram with longitudinal structural equation modelling
Source: Int J Equity Health. 2024 May 1;23:87. doi: 10.1186/s12939-024-02172-w (PMC11061969; doi:10.1186/s12939-024-02172-w)
Supplement: Supplementary file 1 — Supplementary Material 1. [file 12939_2024_2172_MOESM1_ESM.docx]

**Additional file 1: Further methodological considerations**

Below, several more methodological points are discussed. These correspond with specific steps in the *“Steps to a quantified model”* part of the methods section of the paper, but were deemed (by us, the authors) to be too extensive and/or technical to be included in the main text. Because we do believe all of these considerations to be essential to someone who aims to conduct this kind of study, we want to provide the reader with additional information here, with useful references to literature that provides full coverage of the topics. Note: the considerations below are *only* the aspects that were not yet described in the main text.

*Simplifying the CLD (Step 1)*

It is worth noting that omitting or merging variables does not necessarily mean that their effects are filtered out of the model: they can still be implicitly present in mechanisms. For example, in the CLD of Figure 1, population health leads to healthy behaviour via health skills. ‘Health skills’ has no function other than this mediating role, so if omitted, its effect is still implicitly present in the effect from health on healthy behaviour. It is however important to base such simplifications on the theoretical expectation that the variable to be omitted has a mediating effect within the mechanisms so that it does not become an unmeasured confounder.

*Measurement models and confirmatory factor analysis (Step 2)*

As is usual in SEM, confirmatory factor analysis (CFA) was used in this study to infer latent variables from multiple observed indicators [1]. A set of indicators that are theoretically expected to measure the latent construct – such as the Mental Health Inventory 5 (MHI-5) indicators for mental health [2], which were used in this study – are tested for their relationships to an underlying latent variable. These tests consist of checking the height of the factor loadings (the degree of association between the latent variable and an observed indicator) and of overall model fit statistics [3]. If the relationships between observed indicators and the underlying latent variable are solid enough, the latent variable can then be used in a structural model as if it were an ‘ordinary’ continuous variable. In this study, these tests showed that indicators for mental health and physical health or capability did not correspond with just one, but two underlying latent variables for health. Therefore, the variables of mental health and physical capability were included as two separate latent constructs.

*Operationalisation of the latent variable of physical health (Step 2)*

For physical health, multiple options for operationalisation were considered. However, using a single-indicator operationalisation would be overly restrictive and susceptible to measurement error, while chronic disease was also far removed from the meaning of health as used by the stakeholders who built the CLD (whose description came closer to ‘health as wellbeing’). Additionally and more practically, chronic disease items were not suitable for constructing a multi-item latent variable and including the ‘number of chronic illnesses’ or the (dichotomous) presence of chronic illness would be problematic because it provides limited information. A dichotomous endogenous variable would also cause a problem with threshold invariance over time: that it is not a testable assumption that the categories have the same threshold separating the two outcome categories at different time points. A minimum of two thresholds – so three outcome categories – would be required in order to test this assumption. Given that the four indicators of physical capability (or activities of daily living) did load on the same latent construct well, it was decided to limit the scope of the variable and include it under the name ‘physical capability’.

*Scales of the latent variables (Step 2)*

Latent variables, such as mental health and physical capability in this study, do not have their own scales. Instead, in order to attain model identification and for the latent variables to obtain scales, a scaling method has to be chosen [4, 5]. In this study, the factor loadings of the first observed indicator (the one with the highest factor loading) were set to 1, for both mental health and physical capability. These indicators are ‘feeling down’ and ‘walking up a flight of stairs’, respectively. Additionally, the intercepts for both latent variables are set to 0 for the first time point (while the other four time points are freely estimated). This scaling method has a number of different names, such as the marker variable approach, referent intercept identification, or fixed marker scaling [4-6]. By using this approach, the latent variables get their scales based on that of the ‘marker variable’. The correct way to interpret regression coefficients for effects between two latent variables is as follows: “*When the initial endogenous latent variable changes in such a way that its referent indicator changes by one unit, then the target latent variable changes in such a way that its referent indicator changes by* [*β*] *units.*” [4], p. 193, where β is the regression coefficient. However, it should be kept in mind that the *means* of the latent variables (the factor scores) are very much arbitrary and are not also interpretable in terms of the referent indicator.

*Measurement invariance (Step 2)*

It is important that the metric (or scale – the two terms are often used interchangeably) of a latent variable stays the same at each time point. This means that the possible observed values for a respondent do not change (for example, 0 to 10 in one year and 1 to 10 in another year), but also that the values themselves have the same meaning over time. For example, saying you are happy ‘often’ in 2015 has to *mean* the same thing to respondents as it does in 2019, or else any change that is detected may be because something else is measured. One reason why values’ meanings change over time may be that the wording of a survey question was changed [6], which is relatively easy to detect, but other reasons such as a cultural changes affecting interpretation could also be responsible. Measurement invariance can be tested in multiple steps: configural (model form), metric (factor loadings), scalar (item intercepts and thresholds), and uniqueness invariance (residuals) [6-8]. A cross-lagged panel model, such as used in this study, requires metric invariance. Invariance testing, like all the other model fit tests in this study, was done comparing models based on the commonly-used fit indices CFI, TLI, RMSEA, and SRMR [6-9]. Chi-square (difference) tests were not conducted, as these are very sensitive to large sample sizes [9], such as the one used in this study. The assumption of metric measurement invariance held, based on the tests.

*Choosing an estimator (step 3)*

Choosing a suitable estimator depends on the characteristics of the data. If a model has ordinal endogenous observed variables (even if they are indicators for latent variables) – as is the case in this study – opting for a weighted least squares estimator is a good choice [10]. Conversely, if one has a model that includes only continuous variables (and somewhat normal distributions), it makes more sense to use maximum likelihood (or robust maximum likelihood), as this estimator can use full information maximum likelihood, which handles missing data in a very efficient way [11]. The default weighted least squares estimator in the R software package ‘*lavaan’* (used in this study) is ‘weighted least squares with mean and variance adjusted’ (abbreviated as WLSMV) [12, 13], which is the estimator that was selected for this study. This estimator was combined with pairwise deletion to handle missing data.

*Cross-lagged panel models (Step 4)*

In a CLPM, endogenous variables are influenced by themselves and other variables at an earlier time point. The CLPM circumvents the issue of simultaneity (having reciprocal effects, the feedback loops in the CLD) by treating every variable at every time point as a separate variable. There is not just one variable for mental health, for example, but a variable for each one of the five time points of the model. This way, reciprocal relationships can be modelled without having simultaneity. In a CLPM, the distance between time points is fixed. For example, in the present study this time interval is one year. Autoregressions can be seen as a measure of inertia: to what extent is a variable dependent on its own previous value? Cross-lags are interpreted as the extent to which variables are dependent on the previous value of a different variable. All the regressions and covariances between variables together make up the SEM’s structural part.

*Equality constraints on regression coefficients (Step 4)*

Regression coefficients can be constrained to equality at different time points. Doing so has the advantage that every effect is simplified into one coefficient, instead of one coefficient for every particular period (e.g., from 2015 to 2016). This not only makes it much easier to interpret the coefficients, but it often also makes theoretical sense to expect that one variable influences another in the same way from year to year. Constraining to equality means that the two arrows in Fig. 6 of the main paper that are labelled *a* will have the same coefficient, and the same for *b*, *c*, and *d*. Testing whether adding these additional constraints to the model is reasonable can be tested in the same way as testing measurement invariance.

*Endogenous/exogenous and level of measurement (Step 4)*

Whether a variable is endogenous or exogenous depends on the structural model and the level of measurement has different consequences for both sorts. In both a SEM and in a CLD, many or even all variables can be endogenous, while simultaneously also influencing other variables. Endogenous variables in a longitudinal SEM are usually measured at multiple time points and the level of measurement can be continuous or ordinal. Endogenous variables with discrete outcome values pose additional challenges for estimation and interpretation [14]. It is most convenient if all endogenous variables are measured on a continuous scale. Nominal variables are particularly difficult (and will not be further elaborated upon in this paper), but ordinal endogenous variables are still very usable. They just require a different estimator than continuous data (WLSMV instead of maximum likelihood) and the assumption that there is a continuous and normally distributed scale underlying the variable’s discrete values [6, 7]. By putting the categories’ thresholds on a normal curve, it is possible to infer this underlying continuous variable, called y*, which the model can deal with. The thresholds of longitudinal ordinal endogenous variables also require invariance testing [15].

Exogenous variables can be measured at multiple time points or at only one time point. If an exogenous variable is binary, it can simply be treated as continuous. Ordinal variables with more than two categories can be included as multiple (k – 1) binary variables. Sex and migration background are binary exogenous variables. Furthermore, all three exogenous (‘control’) variables included in this study (age, sex, and migration background) are time invariant, meaning they stay the same for each individual over time and therefore only one measurement is used for each of these variables.

*Indirect effect paths and significance (Step 5)*

For indirect effects to exist between two variables, it is not necessary for a direct effect to exist as well. Moreover, non-significant direct paths are also included in the calculations of indirect effects; it is only necessary for the path of an indirect effect as a whole to be significant for it to be included in the total effect [16]. Individual indirect effects can be very small, but are next to always statistically significant; the difference between counting only significant effects or all effects is negligible. The question is therefore not whether an effect is statistically significant, but whether the effect size is substantial [17].

*Standardised coefficients*

Standardised coefficients are derived by multiplying the unstandardised coefficients by the standard deviation of X divided by the standard deviation of Y, meaning that they are expressed in terms of a comparison between the standard deviations. Standardised coefficients are used as a way of cautiously comparing the order of magnitude of effects to those of other effects, though they do not have much in the sense of real-world meaning, and they are all technically still in different units. What constitutes a negligible, small, or large effect depends on interpretation and specific contexts. It is not usually recommended to use only standardised coefficients to try to do so [17], as unstandardised coefficients can often offer a more intuitive interpretation of the size of an effect.

**References**

1. Little TD. **Longitudinal structural equation modeling**: Guilford press; 2013.

2. Berwick DM, Murphy JM, Goldman PA, Ware JE, Barsky AJ, Weinstein MC. **Performance of a Five-Item Mental Health Screening Test**. *Medical Care* 1991, **29**(2):169-176.

3. Mackinnon S, Curtis R, O'Connor R. **A Tutorial in longitudinal measurement invariance and cross-lagged panel models Using lavaan**. *Meta-Psychology* 2022, **6**.

4. Klopp E, Klößner S. **The impact of scaling methods on the properties and interpretation of parameter estimates in structural equation models with latent variables**. *Structural Equation Modeling: A Multidisciplinary Journal* 2021, **28**(2):182-206.

5. Little TD, Slegers DW, Card NA. **A non-arbitrary method of identifying and scaling latent variables in SEM and MACS models**. *Structural equation modeling* 2006, **13**(1):59-72.

6. Newsom JT. **Longitudinal structural equation modeling: A comprehensive introduction**: Routledge; 2015.

7. Bowen NK, Masa RD. **Conducting measurement invariance tests with ordinal data: A guide for social work researchers**. *Journal of the Society for Social Work and Research* 2015, **6**(2):229-249.

8. Putnick DL, Bornstein MH. **Measurement invariance conventions and reporting: The state of the art and future directions for psychological research**. *Developmental review* 2016, **41**:71-90.

9. Meade AW, Johnson EC, Braddy PW. **Power and sensitivity of alternative fit indices in tests of measurement invariance**. *Journal of applied psychology* 2008, **93**(3):568.

10. Flora DB, Curran PJ. **An empirical evaluation of alternative methods of estimation for confirmatory factor analysis with ordinal data**. *Psychological methods* 2004, **9**(4):466.

11. Enders CK, Bandalos DL. **The relative performance of full information maximum likelihood estimation for missing data in structural equation models**. *Structural equation modeling* 2001, **8**(3):430-457.

12. Rosseel Y. **lavaan tutorial**. https://lavaan.ugent.be/tutorial/cat.html. Accessed 19 February 2024.

13. Rosseel Y. **lavaan: An R package for structural equation modeling**. *Journal of statistical software* 2012, **48**:1-36.

14. Grimm KJ, Ram N, Estabrook R. **Growth modeling: Structural equation and multilevel modeling approaches**: Guilford Publications; 2016.

15. Mehta PD, Neale MC, Flay BR. **Squeezing interval change from ordinal panel data: latent growth curves with ordinal outcomes**. *Psychological methods* 2004, **9**(3):301.

16. Zhao X, Lynch Jr JG, Chen Q. **Reconsidering Baron and Kenny: Myths and truths about mediation analysis**. *Journal of consumer research* 2010, **37**(2):197-206.

17. Kline RB. **Beyond significance testing: statistics reform in the behavioral sciences**. Washington, D.C.: American Psychological Association; 2013.
