# Supplementary material for: Quantifying reciprocal relationships between poverty and health: combining a causal loop diagram with longitudinal structural equation modelling
Source: Int J Equity Health. 2024 May 1;23:87. doi: 10.1186/s12939-024-02172-w (PMC11061969; doi:10.1186/s12939-024-02172-w)
Supplement: Supplementary file 2 — Supplementary Material 2. [file 12939_2024_2172_MOESM2_ESM.docx]

**Additional file 2: Overview of observed variables used in the study and their operationalisations**

**Datasets and raw variables used**

CBS data (2016 to 2020, observation took place at 1 January of each year), full codebooks are available on their website [1]:

| **Dataset** | **Variable** | **Description** |
| --- | --- | --- |
|  | RINPERSOON | Unique identifier for each individual |
|  | RINPERSOONHKW | Unique identifier for each household |
| INHATAB | INHARMLAG | Standardised household income expressed in terms of the low income threshold |
| VEHTAB | VEHWVEREXEWH | Household wealth excluding home ownership assets and debts |
| INPATAB | INPT1000WER  INPT1020AMB  INPT1030DGN  INPT1040NAT  INPT1060OVE  INPT2070WIN | All different sources of income in individual basis |
| GBAPERSOONTAB | GBAGEBOORTELANDMOEDER | Country of birth of mother |
|  | GBAGEBOORTELANDVADER | Country of birth of father |
|  | GBAGEBOORTEJAAR | Year of birth respondent |
|  | GBAGESLACHT | Sex (dichotomous, registered as male/female) |

LISS data (2015 to 2019, observation took place around the end of each year), full codebooks are available on their website [2]:

| **Dataset and variable number** | **Variable name** |
| --- | --- |
| *nomem_encr* | Unique identifier for each individual |
| ***Background*** |  |
| woning | Type of dwelling inhabited by household |
| oplmet | Level of education: highest degree attained |
| ***Health (ch)*** |  |
| 001 | Gender (male/female) |
| 002 | Age |
| 011 | Frequency of feeling (in last month): anxious |
| 012 | Frequency of feeling (in last month): down |
| 013 | Frequency of feeling (in last month): calm |
| 014 | Frequency of feeling (in last month): gloomy |
| 015 | Frequency of feeling (in last month): happy |
| 016 | Body height in centimeters |
| 017 | Body weight in kilograms |
| 023 | How much effort it takes to: walk 100 meters |
| 027 | How much effort it takes to: walk up a flight of stairs |
| 031 | How much effort it takes to: carry an object of 5 kilograms |
| 035 | How much effort it takes to: bathe or shower |
| 125 | Has ever smoked (yes/no) |
| 126 | Is smoker now (yes/no) |
| 127 | Smokes or has smoked cigarettes (yes/no) |
| 128 | Smokes or has smoked pipe (yes/no) |
| 129 | Smokes or has smoked cigars (yes/no) |
| 130 | Number of cigarettes per day |
| 131 | Number of pipe per day |
| 132 | Number of cigars per day |
| 133 | How often drank alcohol in last 12 months |
| 134 | Drank alcohol last week (yes/no) |
| 135 | Number of days drank alcohol last week |
| ***Social (cs)*** |  |
| 001 | Satisfaction with amount of leisure time |
| 283 | Satisfaction with social contacts |

**Operationalisation of variables**

***Mental health***

Latent variable, constructed from all items the often-used Mental Health Inventory 5 (MHI-5). They are available in the LISS dataset in the exact form as designed for the MHI-5 (items ch011 to ch015). The observed indicators have six categories, which were coded from 0 to 5, where 0 means the worst and 5 the best mental health. These variables were measured on an ordinal scale. The mental health latent variable derived its scale from the first indicator (the one with the highest factor loading), which is ‘feeling down’. Factor loadings can be found in the raw *lavaan* model output in Additional file 5.

***Physical capability***

Custom-made latent variable, based as closely as possible on four aspects that are part in often-used questionnaires of EQ-5D and SF-12. The variable used are ch023, ch027, ch031, and ch035. Item ch030 was also tested in a CFA, but was omitted because of low factor loadings, meaning it does not seem to fit on the same underlying construct as the other four. The observed indicators have five categories, which were coded from 0 to 4, where 0 means the worst and 4 the best physical capability. These variables were also measured on an ordinal scale. The physical capability latent variable derived its scale from the first indicator (the one with the highest factor loading), which is ‘walking up a flight of stairs’. Factor loadings can be found in the raw *lavaan* model output in Additional file 5.

***Income***

The CBS variable INHARMLAG was used to measure income. This is annual household income, adjusted to account for household composition and expressed in percentages of the low income threshold for that year. This value was then divided by 100 so that a value of 1 corresponds to one time the low income threshold. The minimum registered value was 0.01 (0 to 1 percent of low income threshold) and the maximum registered value was 9.99 (999 percent of that threshold or higher). This variable can be seen as being on a continuous scale.

***Financial wealth***

Variable VEHWVEREXEWH was used to measure financial wealth. This data reflects household assets minus debts, not counting assets and debts relating to ownership of one’s own home (value of the home and mortgage). The reason behind excluding those specific assets and debts is that value of the home is not liquid and cannot easily be used to counter financial setbacks. Discounting these assets, the corresponding debts (mortgages) should also be excluded. Additionally, households with more financial opportunity are likely to also buy more expensive real estate. As a result, completely healthy and affluent households with a high debt in the form of a mortgage could quickly be labelled as having low financial wealth. To limit the effect of extreme outliers, cut-offs were placed, so that the minimum registered value was -50 (times the median wealth) and the maximum registered value was 50 (times the median wealth). This variable was measured on a continuous scale.

***Satisfaction with leisure time; satisfaction with social contacts***

These variables were adopted directly from the LISS data, by simply using items cs001 and cs283. They were measured on an 11-point scale, from 0 to 10, where 0 means “not at all satisfied” and 10 means “completely satisfied”. Both variables were treated as continuous in the analyses.

***Body Mass Index (BMI)***

This was simply calculated with the formula of *body weight / (body height)^2^*, using self-reported data for both. Extreme results under 10 and over 100 were removed, as it can usually be assumed that these are indicative of a faulty measurement. This was measured on a continuous scale.

***Alcohol use***

For this variable, three items were used. The reason for needing items ch133 and ch134 was because respondents that reported not drinking alcohol did not answer the question for ch135 and were otherwise recorded as ‘missing’, which was of course very much not at random. These respondents were given a value of 0. Item ch135 asks *“On how many of the past seven days did you have a drink containing alcohol?”*, resulting in a value between 0 and 7. This variable was treated as continuous in the analyses.

***Smoking***

Items ch125 and ch126 were used for a quite similar reason: to identify respondents who have answered the questions, but indicated that they have never smoked or do not smoke anymore. Questions ch130 to ch132 count the average number of cigarettes, cigars, and pipes that the respondent smokes per day. All of these were summed to get one composite score of average units of tobacco per day. Because a continuous variable would be very skewed due to the high number of respondents with value 0, an ordinal scale was used, with respondents being categorised as 0 ‘no smoking’, 1 ‘smoking up to 5 units per week’, or 2 ‘smoking more than 5 units per week’.

***Exogenous variables***

All exogenous variables in this study are time-invariant, meaning it is fixed and does not vary over the years. However, for the operationalisation of some of the variables, data from multiple years was used, but they were then combined into one variable. Therefore, none of these time-invariant covariates are longitudinal (as the name also reflects).

*Age, sex, and migration background*

Age was operationalised as age in 2015. All available data were used for this, from both CBS and LISS. Age from CBS data was obtained by doing ‘2015 – year of birth’ with 2019 data. This number was compared with all available LISS data points and the average difference was subtracted from CBS data. In the few cases where CBS age data was not available, the average age from LISS data was used. Consequently, there are no missing data for this variable.

Sex (male or female) was included as a binary variable. This is how the variable was recorded in both datasets and it easier to model and interpret than an ordinal variable. In the few cases in which there were conflicting measurements over the years in the variable sex, the latest available value was used. Similarly, if LISS and CBS data conflicted, LISS data were used, as this was what the respondent indicated themselves.

Migration background was constructed from data on the country of origin of both parents and a dichotomous variable was computed. Respondents with one or both parents who were born in another country than The Netherlands are coded as having a migration background. Respondents whose parents were both born in The Netherlands are coded as not having a migration background.

*Work status, level of education, and home ownership*

All three of the variables that were included as exogenous in the sensitivity analyses (work status, level of education, home ownership) are dichotomous. Having paid work was operationalised as personally having income from paid work that amounts to at least 12 hours [3] of minimum-wage income, for at least most of the observed years. This income data was taken from CBS. For level of education, LISS data were used, with a distinction made between ‘highly educated’ [4] and ‘other’, taken at the latest measurement available. Home ownership was also operationalised with LISS data, as living in a self-owned home or not.

**References**

1. CBS. **Catalogus microdata**. https://www.cbs.nl/nl-nl/onze-diensten/maatwerk-en-microdata/microdata-zelf-onderzoek-doen/catalogus-microdata. Accessed 23 Febraury 2024.

2. Centerdata. **LISS panel**. https://www.centerdata.nl/en/liss-panel. Accessed 19 February 2024.

3. CBS. **Labour force (12-hours threshold)**. https://www.cbs.nl/en-gb/our-services/methods/definitions/labour-force--12-hours-threshold--. Accessed 23 February 2024.

4. CBS. **Opleidingsniveau**. https://www.cbs.nl/nl-nl/nieuws/2019/33/verschil-levensverwachting-hoog-en-laagopgeleid-groeit/opleidingsniveau. Accessed 23 February 2024.
